# A189 EMERGENCY DEPARTMENT UTILIZATION AND RISK OF INTESTINAL RESECTION IS LOWER AMONG CHILDREN DIAGNOSED WITH INFLAMMATORY BOWEL DISEASE BEFORE 10 YEARS OF AGE: A MULTIPROVINCE POPULATION-BASED COHORT STUDY

**DOI:** 10.1093/jcag/gwac036.189

**Published:** 2023-03-07

**Authors:** E Kuenzig, H Singh, A Bitton, G G Kaplan, M W Carroll, A R Otley, T A Stukel, S Spruin, A M Griffiths, D R Mack, K Jacobson, G C Nguyen, L E Targownik, W El-Matary, E I Benchimol

**Affiliations:** 1 Child Health Evaluative Sciences, SickKids Research Institute; 2 SickKids Inflammatory Bowel Disease Centre, Division of Gastroenterology, Hepatology and Nutrition, The Hospital for Sick Children, Toronto; 3 University of Manitoba IBD Clinical and Research Centre; 4 Department of Internal Medicine, Max Rady College of Medicine, , University of Manitoba; 5 Research Institute at CancerCare Manitoba, Winnipeg; 6 Gastroenterology and Hepatology, McGill University Health Centre, Montreal; 7 Medicine & Community Health Sciences, University of Calgary, Calgary; 8 Pediatrics, University of Alberta, Edmonton; 9 Pediatrics, Dalhousie University, Halifax; 10 ICES; 11 Institute of Health Policy, Management and Evaluation; 12 Paediatrics, University of Toronto, Toronto; 13 Pediatrics, University of Ottawa; 14 CHEO Inflammatory Bowel Disease Centre, Division of Gastroenterology, Hepatology and Nutrition, CHEO; 15 CHEO Research Institute, Ottawa; 16 Department of Pediatrics, BC Children's Hospital Research Institute, University of British Columbia, Vancouver; 17 Mount Sinai Hospital Centre for Inflammatory Bowel Disease, Department of Medicine, University of Toronto, Toronto; 18 Pediatrics, University of Manitoba, Winnipeg, Canada

## Abstract

**Background:**

In Canada, the incidence of inflammatory bowel disease (IBD) is increasing faster among those <10 years (y) of age than in any other age group. Understanding the health services burden of IBD in this population is important for health system planning.

**Purpose:**

To compare healthcare utilization and risk of surgery among children diagnosed with IBD across age groups defined by the Paris Classification (A1a: <10y; A1b: 10 to <16y) across 5 Canadian provinces.

**Method:**

Children diagnosed with IBD <16 years of age were identified from health administrative data using validated algorithms in Alberta, Manitoba, Nova Scotia, Ontario, and Québec. Negative binomial regression models were used to compare (1) the pre-diagnosis frequency of health services utilization (outpatient, emergency department (ED), and hospitalization) using diagnostic codes suggestive of future IBD and (2) the annual post-diagnosis frequency of IBD-specific and IBD-related (signs, symptoms, and extra-intestinal manifestations of IBD) visits among children diagnosed <10y (A1a) and 10 to <16y (A1b). Cox proportional hazard models compared the risk of surgery (identified with validated procedure codes) across age groups. All regression models were adjusted for sex, rural/urban residence, and mean neighbourhood income quintile. Province-specific event counts (all ages combined) and models (comparing age groups; reference: A1b [10 to <16y]) were pooled using random-effects meta-analysis.

**Result(s):**

Among 5124 children with IBD (1165 [23%] were <10y at diagnosis), the mean number of pre-diagnosis healthcare encounters was 1.0 (95% CI 0.38 to 1.68, I^2^=99.6%). The mean annual post-diagnosis number of IBD-specific outpatient visits was 3.2 (95% CI 1.9-4.4, I^2^=99.6%); hospitalizations, 0.19 (95% CI 0.17-0.21, I^2^=74%); ED visits, 0.17 (95% CI 0.19-0.39, I^2^=99%). The mean annual post-diagnosis number of IBD-related outpatient visits was 3.9 (95% CI 2.3-5.5, I^2^=99.7%); hospitalizations, 0.21 (95% CI 0.19-0.23, I^2^=79%); ED visits, 0.29 (95% CI 0.19-0.39, I^2^=97%). Intestinal resection or colectomy within 5y of diagnosis occurred in 13% (95%CI 8-22, I^2^=93%) with Crohn’s disease (CD) and 16% (95% CI 14-18, I^2^=40%) with ulcerative colitis. IBD-specific ED visits (RR 0.70, 95% CI 0.50-0.97, I^2^=80) and the risk of intestinal resection in CD (HR 0.49, 95% CI 0.26-0.92, I^2^=40%) were significantly lower among children diagnosed <10y. There were no age-related differences in pre-diagnosis health services utilization or other post-diagnosis outcomes, including frequency of outpatient visits to a gastroenterologist.

**Conclusion(s):**

Health services utilization was generally similar for children diagnosed with IBD at <10y and between 10 and <16y, except for lower rates of IBD-specific ED visits and intestinal resection in children with CD. Further exploration of between-province differences, represented by the high statistical heterogeneity (I^2^) in the meta-analyses, is needed to understand sources of variation in care.

**Please acknowledge all funding agencies by checking the applicable boxes below:**

CCC

**Disclosure of Interest:**

E. Kuenzig: None Declared, H. Singh Consultant of: Amgen Canada, Bristol-Myers Squibb Canada, Sandoz Canada, Roche Canada, Takeda Canada and Guardant Health, A. Bitton: None Declared, G. Kaplan Grant / Research support from: Ferring, Consultant of: AbbVie, Janssen, Pfizer, Amgen, Sandoz, Pendophram, and Takeda, Speakers bureau of: AbbVie, Janssen, Pfizer, Amgen, Sandoz, Pendophram, and Takeda, M. Carroll: None Declared, A. Otley Grant / Research support from: Research support: AbbVie Global. Research site: AbbVie, Pfizer, Eli-Lily, Janssen, Consultant of: AbbVie Canada, T. Stukel: None Declared, S. Spruin: None Declared, A. Griffiths Grant / Research support from: Abbvie, Consultant of: Abbvie, Amgen, BristolMyersSquibb, Janssen, Lilly, Takeda, Speakers bureau of: Abbvie, Janssen, Takeda, D. Mack: None Declared, K. Jacobson Grant / Research support from: Abbvie Canada and Janssen Canada, Consultant of: Abbvie Canada, Janssen Canada, Merck Canada and Mylan Pharmaceuticals, Speakers bureau of: Abbvie Canada and Janssen Canada, G. Nguyen: None Declared, L. Targownik Grant / Research support from: Janssen Canada, Consultant of: AbbVie Canada, Sandoz Canada, Takeda Canada, Merck Canada, Pfizer Canada, Janssen Canada, and Roche Canada, W. El-Matary Consultant of: Abbvie and MERCK, Speakers bureau of: Abbvie and MERCK, E. Benchimol Consultant of: McKesson Canada, Dairy Farmers of Ontario (unrelated to medications used to treat inflammatory bowel disease)

